# Evolution of blue-flowered species of genus *Linum* based on high-throughput sequencing of ribosomal RNA genes

**DOI:** 10.1186/s12862-017-1105-x

**Published:** 2017-12-28

**Authors:** Nadezhda L. Bolsheva, Nataliya V. Melnikova, Ilya V. Kirov, Anna S. Speranskaya, Anastasia A. Krinitsina, Alexey A. Dmitriev, Maxim S. Belenikin, George S. Krasnov, Valentina A. Lakunina, Anastasiya V. Snezhkina, Tatiana A. Rozhmina, Tatiana E. Samatadze, Olga Yu. Yurkevich, Svyatoslav A. Zoshchuk, Аlexandra V. Amosova, Anna V. Kudryavtseva, Olga V. Muravenko

**Affiliations:** 1 Engelhardt Institute of Molecular Biology, Russian Academy of Sciences, Moscow, Russia; 2 Shemyakin-Ovchinnikov Institute of Bioorganic Chemistry, Russian Academy of Sciences, Moscow, Russia; 3 Faculty of Biology, Lomonosov Moscow State University, Moscow, Russia; 4 All-Russian Research Institute for Flax, Torzhok, Russia

**Keywords:** Flax, Phylogeny, rRNA genes, High-throughput sequencing, Karyotype, FISH

## Abstract

**Background:**

The species relationships within the genus *Linum* have already been studied several times by means of different molecular and phylogenetic approaches. Nevertheless, a number of ambiguities in phylogeny of *Linum* still remain unresolved. In particular, the species relationships within the sections *Stellerolinum* and *Dasylinum* need further clarification. Also, the question of independence of the species of the section *Adenolinum* still remains unanswered. Moreover, the relationships of *L. narbonense* and other species of the section *Linum* require further clarification. Additionally, the origin of tetraploid species of the section *Linum* (2n = 30) including the cultivated species *L. usitatissimum* has not been explored. The present study examines the phylogeny of blue-flowered species of *Linum* by comparisons of 5S rRNA gene sequences as well as ITS1 and ITS2 sequences of 35S rRNA genes.

**Results:**

High-throughput sequencing has been used for analysis of multicopy rRNA gene families. In addition to the molecular phylogenetic analysis, the number and chromosomal localization of 5S and 35S rDNA sites has been determined by FISH.

Our findings confirm that *L. stelleroides* forms a basal branch from the clade of blue-flowered flaxes which is independent of the branch formed by species of the sect. *Dasylinum*. The current molecular phylogenetic approaches, the cytogenetic analysis as well as different genomic DNA fingerprinting methods applied previously did not discriminate certain species within the sect. *Adenolinum.* The allotetraploid cultivated species *L. usitatissimum* and its wild ancestor *L. angustifolium* (2n = 30) could originate either as the result of hybridization of two diploid species (2n = 16) related to the modern *L. gandiflorum* and *L. decumbens*, or hybridization of a diploid species (2n = 16) and a diploid ancestor of modern *L. narbonense* (2n = 14).

**Conclusions:**

High-throughput sequencing of multicopy rRNA gene families allowed us to make several adjustments to the phylogeny of blue-flowered flax species and also reveal intra- and interspecific divergence of the rRNA gene sequences.

**Electronic supplementary material:**

The online version of this article (doi: 10.1186/s12862-017-1105-x) contains supplementary material, which is available to authorized users.

## Background

The genus *Linum* (*Linacea*), which involves about 200 species, is subdivided into a group of sections based on morphological traits [1–3]. The investigation of the phylogeny of the genus *Linum* has been performed several times by means of different molecular and phylogenetic approaches. Particularly, due to the similar results obtained by AFLP, RAPD and SSAP methods, the members of the genus could be subdivided into the groups of species with the similar or even identical karyotype structure [4–7]. In some instances, the groups species revealed according to the genome similarities coincide with the separate sections of the genus with the exception of the sect. *Linum* and *Linastrum* involving several groups. Unfortunately, DNA fingerprinting is not an adequate method for studying the phylogenetic relationships between the separate groups of species. Mostly, these relationships were revealed by molecular phylogenetic investigations performed with the use of nuclear ibosomal ITS1 and ITS2 and plastid sequences of flax [8]. The results of these studies showed that the genus *Linum* was not monophyletic but combined the members of two sister clades: yellow-flowered and blue-flowered flaxes. Later, the analysis of the transcriptomes of 11 flax species representing different sections was performed with the use of high-throughput sequencing [9]. Based on the analysis of 413 nuclear genes, the phylogeny of these 11 species was constructed, and it was almost identical to *Linum* phylogeny created with the use of nuclear ribosomal ITS and chloroplast genes [8]. The similar results were obtained by multiplexed shotgun sequencing of a great number of nuclear and chloroplast genes of 16 flax species [10]. Thus, the phylogenetic relationships within the genus *Linum* have been adequately investigated, however, a number of details are still unresolved. Particularly, the species relationships within the monotypic sect. *Stellerolinum* and the sect. *Dasylinum* need further clarification. According to the phylogeny of *rbcL*, *L. stelleroides* (sect. *Stellerolinum*) is a sister to sect. *Dasylinum*. Besides, the analysis of ITS and other chloroplast data showed that this species is a sister to *Dasylinum* plus the remaining members of sect. *Linum* including the *L. perenne* group [8]. The phylogeny of the sect. *Adenolinum* (syn. *L. perenne* group) also needs some refinements. Based on the morphological traits, about 20 species can be separated within the sect. *Adenolinum*. However, this identification of the species are not unambiguous as the morphological traits are mainly the quantitative characters which depend on the environment conditions [11].

It was found that all members of the section have similar karyotypes. Moreover, genomic differences among the certain species of the section were not revealed by AFLP, RAPD or SSAP methods [4, 5, 7]. As the analysis of the sequences of nuclear and cytoplasmic genes was performed only for a limited number of species [8–10], it is not possible to estimate accurately the intra- or interspecific divergence of these sequences among the members of the sect. *Adenolinum*, and the question of species independence within this section still remains unanswered.

In the present study, we investigated the relationships within the clade of blue-flowered flaxes based on the analysis of ITS1-5.8S-ITS2 sequences of 35S rRNA genes and also 5S rRNA genes. Moreover, in angiosperm genomes, 35S and 5S rRNA genes are usually localized separately as multiple tandem repeats. The coding sequences of rRNA genes are highly conserved unlike the sequences of non-coding spacers which are polymorphic and therefore, can be widely used in phylogenetic studies [12, 13]. The distinct advantage of using of 5S rRNA genes as molecular markers in phylogenetic studies is the fact that these genes are inherited from the both parent species in hybrid plants. In contrast, 35S rRNA genes of one of the parents can be eliminated in the hybrid genome [14, 15]. For analysis of the selected gene sequences, we used the high-throughput target sequencing. This is a more adequate technique for estimation of intraspecific variability and, consequently, a degree of interspecific differences compared to the traditional approach which is based on the sequencing of few cloned sequences. For clarification of the phylogenetic relationships within the sect. *Adenolinum*, we increased significantly the number of the studied species and specimens. Additionally, for all the examined plant samples, the karyotypic studies and chromosome mapping of 5S and 35S rRNA genes were carried out.

## Methods

### Plant material

In the present study, 43 flax accessions belonging to 23 species and subspecies from the sections *Linum*, *Adenolinum*, *Dasylinum*, *Stellerolinum* and *Syllinum* were used (Additional file 1). Most of these accessions were obtained from the genebank of Leibniz Institute of Plant Genetics and Crop Plant Research (IPK) (Gatersleben, Germany) and the seed collection of All-Russian Flax Institute (VNIIL) (Torzhok, Russian Federation). The accessions of *L. amurense* and *L. stelleroides* were kindly provided by Dr. L.N. Mironova, Botanic Garden Institute of the Far-Eastern Branch of the Russian Academy of Sciences (BGI of RAS) (Vladivostok, Russian Federation). Several accessions were collected from natural populations by Dr. A.A. Svetlova, Komarov Botanical Institute RAS (St. Petersburg, Russian Federation), by Dr. N.L. Bolsheva, Engelhardt Institute of Molecular Biology RAS (Moscow, Russian Federation), and by Dr. M. Pavelka, Euroseeds (Novy Jicin, Chech Republic).

The species determination of some accessions of wild *Linum* species was performed previously [5–7, 16].

### Chromosome preparation and FISH

Chromosome preparation was carried out according to [17]. FISH with 5S and 35S rDNA probes was performed as described previously [18].

### Preparation and sequencing of DNA libraries

Total DNA was extracted from flax leaves as described earlier [7].

The sequences of 5S rDNA and ITS1-5.8S rDNA-ITS2 were amplified using the method of two-step tailed PCR procedure for library preparations [19]. Primer sequences are shown in Table 1. Before PCR, DNA concentrations were standardized to 10 ng/μl. The first stage of PCR was carried out in total volume of 25 μl containing 1× KAPA2G buffer A (Kapa Biosystems, USA), 0.12 μM of each primer, 250 μM each dNTP (Thermo Scientific, USA), 0.5 U of KAPA2G Fast HotStart DNA Polymerase (Kapa Biosystems) and 25 ng of template DNA. The first-stage PCR primers containing universal tail sequences are shown in Table 1. The PCR conditions were: the initial denaturing step at 95 °C for 1 min followed by 5 cycles of 30 s at 95 °C for 2 min followed by 20 cycles of 15 s at 95 °C, 30 s at 58 °C and 15 s at 72 °C. The final elongation was set for 3 min at 72 °C. The final elongation was set for 5 min at 72 °C. The second-stage PCR was performed with the primers containing barcodes (MIDs) and sequence adapters which converted amplicons into a library for using 454 GS Junior Systems (see Table 1). Amplification was carried out in 25 μl of PCR mix containing 1× KAPA2G buffer A (Kapa Biosystems), 250 μM each dNTP (Thermo Scientific, USA), 0.12 μM of MIDх-454 forward and reverse primers, 0.5 U of KAPA2G Fast HotStart DNA Polymerase (Kapa Biosystems), and 3 μl of the diluted (1:20) first-stage PCR product. The program of amplification for the second stage was 95 °C for 2 min, 10 cycles (95 °C for 15 s, 58 °C for 30 s, 72 °C for 15 s) and 72 °C for 3 min.

**Table 1:** Primer sequences

First-stage PCR specific primers	
5S-F	5’-**TTTCCCAGTCACGACGTT**ATGCACCGGATCCCATCAGA-3′
5S-R	5’-**TAATACGACTCACTATAGGG**AGTGCTGGTATGATCGCACC-3′
ITS-F	5’-**TTTCCCAGTCACGACGTT**TCCTCCGCTTATTGATATGC-3′
ITS-R	5’-**TAATACGACTCACTATAGGG**TCGTAACAAGGTTCCCGTAGGTG-3′
Second-stage PCR primers [19]	
MIDх-454-F	5’-GCCTCCCTCGCGCCATCAG-*MIDx*-**TTTCCCAGTCACGACGTT**-3’
MIDх-454-R	5’-GCCTTGCCAGCCCGCTCAG-*MIDx*-**TAATACGACTCACTATAGGG**-3’

*Note:* Universal tail sequences are marked in bold

The PCR products were separated in 1.5% agarose gel for 5 h at 45 V using ТВЕ buffer and then stained with ethidium bromide. The Bio-Rad Gel Doc system was used for visual detection of the presence of the PCR products. Bands of the expected sizes (~350 bp for 5S rDNA and ~950 for ITS1-5.8S rDNA-ITS2) were excised from agarose gel and purified using Agencourt AMPure beads (Beckman Coulter, USA). Purified amplicon libraries concentration was evaluated using Quant-iT PicoGreen dsDNA Assay Kit (Life technologies, USA) with the use of QuantiFlour fluorometer (Promega, USA). Then, equimolar pooling of barcoded amplicons for 5S rDNA and ITS1-5.8S rDNA-ITS2 libraries was performed and library quality was evaluated using Agilent 2100 Bioanalyzer (Agilent Technologies, USA). After library dilution, emulsion PCR for Lib-A library and 454 sequencing by GS Junior were performed according to the protocols provided by the manufacturer (Roche, Switzerland).

### Phylogenetic analysis and statistical evaluation

The obtained reads of each accession were aligned and then clustered with the use of Allele Builder (http://sourceforge.net/p/allelebuilder). For the clusters representing more than 5% of the obtained reads, consensus sequences were constructed using the program CLC Genomics Workbench v6.5 (http://www.clcbio.com). The alignment of the obtained consensus sequences was performed using the program Clustal W [20]. For ITS1-5.8S rDNA-ITS2 sequences, the parameters for Pairwise Sequence Alignment were: Gap Opening Penalty - 15, Gap Extension Penalty - 6.66; the parameters for Multiple Sequence Alignment were: Gap Opening Penalty - 15; Gap Extension Penalty - 6.66; DNA Weight Matrix - JUB; Transition Weight - 0.5; Delay Divergent Cutoff - 50%. For 5S rDNA, the parameters for Pairwise Sequence Alignment were: Gap Opening Penalty - 15, Gap Extension Penalty - 6.66; parameters for Multiple Sequence Alignment were: Gap Opening Penalty - 12; Gap Extension Penalty - 6.66; DNA Weight Matrix - JUB; Transition Weight - 0.4; Delay Divergent Cutoff −70%.

Phylogenetic analysis was performed using Maximum Likelihood (ML) by PhyML software [21]. The best fit model of nucleotide substitution was determined by JModelTest 2.1.1.0 software [22]. The selected model was YIM3ef + G and TPM3 + I + G for ITS and 5S regions, respectively, established by corrected Akaike Information Criterion (AICc) and Bayesian Information Criterion (BIC). The tree was visualized by iTOL tool [23]. The node support was assessed by the Bayesian-like modification of the approximate likelihood ratio test (aBayes) [24]. Statistical calculations were conducted in MEGA version 6 [25].

## Results

### Karyotype structure and chromosomal mapping of rRNA genes

In the present study, we describe mostly the results of cytogenetic studies of the specimens which have not been published yet as the karyotype structure and localization of ribosomal genes of many flax species had been previously published [5, 6, 16].

**Fig. 1:**
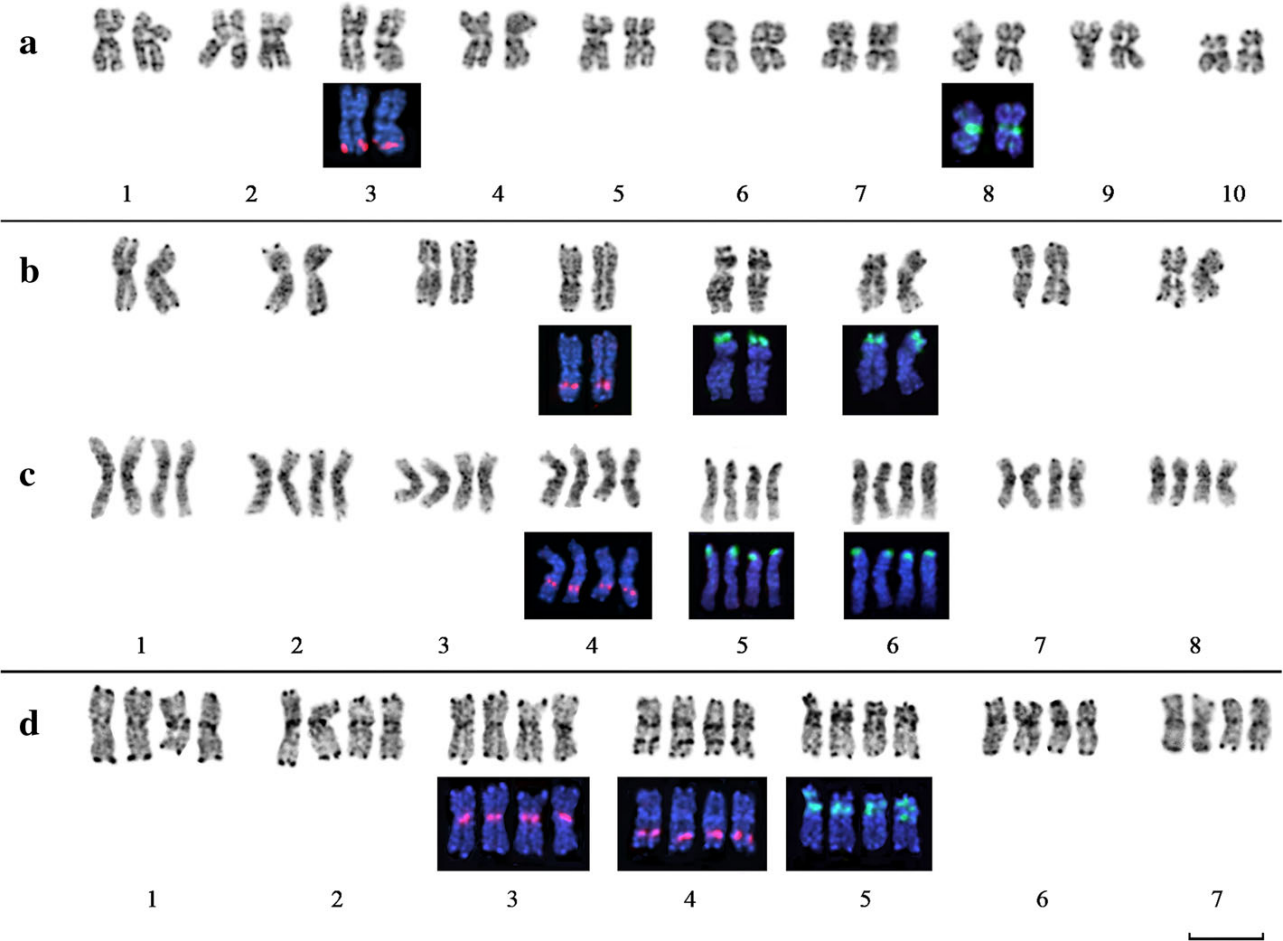
Karyotypes of blue-flowered flaxes with relatively large chromosomes. **a** – *L. stelleroides* (sect. *Stellerolinum*); **b, c** – members of sect. *Dasylinum*: diploid *L. hirsutum* subsp.*hirsutum* (**b**) and tetraploid *L. hirsutum* subsp. *anatolicum;* **d** – *L. narbonense* (sect.*Linum*). Chromosomes are arranged according to their sizes. Inverted DAPI- staining of chromosomes are shown in shades of gray. The positions of rDNA loci revealed by FISH are shown in colored insertions bellow the karyotypes: 35S rDNA – green signals and 5S rDNA – red signals. Scale bar – 5 μM

Our studies showed that *L. stelleroides* (2n = 20), which is the only member of the section *Stellerolinum*, possessed rather large chromosomes (2.5-5 μm). The only site of 35S rRNA genes was revealed in the pericentromeric region of the short arm of chromosome 8, and 5S rDNA site was detected in the distal region of the long arm of chromosome 3 (Fig. 1a).

The members of the section *Dasylinum* had the largest chromosomes found in flax (3.5-6 μm). Among three studied specimens, *L. hirsutum* ssp. *hirsutum* was diploid with 2n = 16, whereas *L. hirsutum* ssp. anatolicum and *L. hirsutum* ssp. *pseudoanatolicum* were tetraploid (2n = 32) containing two similar sets of chromosomes. In genomes of these plants, two loci of 35S rDNA were detected in the distal regions of the short arms of chromosomes 1 and 5, and one locus of 5S rDNA was found in the median region of the short arm of chromosome 4 (Fig. 1b, c).

**Fig. 2:**
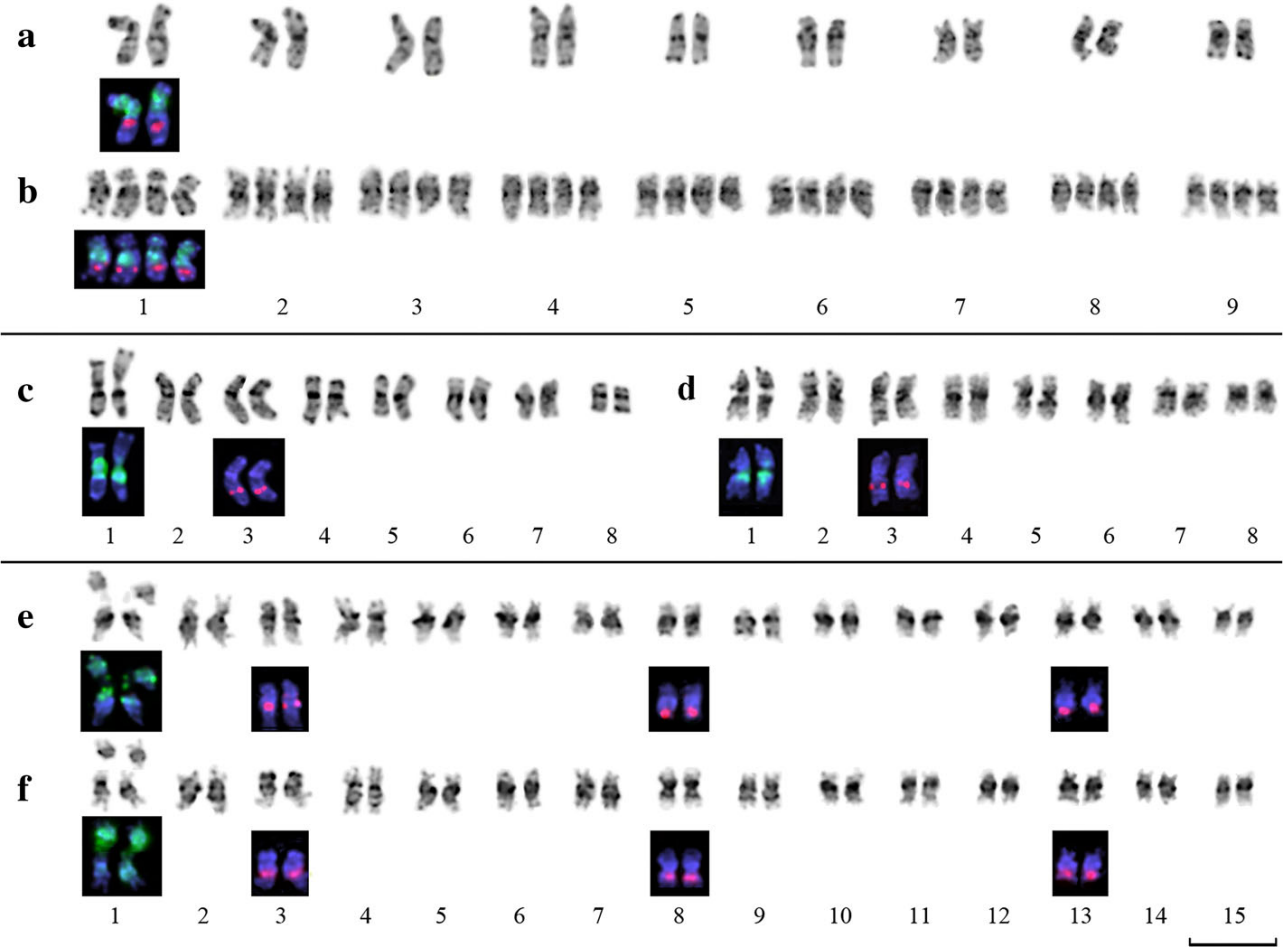
Karyotypes of blue-flowered flaxes with relatively small chromosomes. **a, b** – members of sect. *Adenolinum*: diploid *L. perenne* (**a**) and tetraploid *L. perenne* subsp. *extraaxillare* (**b**); **c**, **d** – members of sect. *Linum* with 2n = 16: *L. grandiflorum* (**c**) and *L. decumbens* (**d**); **e**, **f** – members of sect. *Linum* with 2n = 30: *L. usitatissimum* (**e**) and *L. angustifolium* (**f**). Chromosomes arranged according to their sizes. Inverted DAPI- staining of chromosomes are shown in shades of gray. The positions of rDNA loci revealed by FISH are shown in colored insertions below the karyotypes: 35S rDNA – green signals and 5S rDNA – red signals. Scale bar – 5 μM

All members of the section *Adenolinum* have the similar karyotype structure. The size of chromosomes ranged from 2 to 4 μm. Most of studied samples were diploid with 2n = 18. The specimens *L. perene* K5500, *L. perene* ssp. *anglicum* and *L. perene* ssp. *extraaxilare* were tetraploid with 2n = 36. The 35S rDNA locus was revealed in the pericentromeric region of the short arm of chromosome 1, and 5S rDNA locus was localized in the median region of the long arm of the same chromosome (Fig. 2a, b).

The karyotype of *L. narbonense* possessed two similar chromosome sets and was probably tetraploid with 2n = 4× = 28. The sizes of chromosomes of this species were within 3-5 μm range. The clusters of 35S rDNA were localized in the proximal regions of short arms of two homeologous pairs of chromosomes. Large 5S rDNA clusters were found in the distal regions of two chromosome pairs and also in the pericentromeric heterochromatic regions of two pairs of metacentric chromosomes (Fig. 1d). In some metaphase cells, minor sites of 5S rDNA were detected in the pericentromeric heterochromatin of 2-4 chromosome pairs.

Closely related species *L. grandiflorum* and *L. decumbens* (2n = 16) had the similar structure of karyotypes, but their chromosomes were smaller (2-3.5 μm) than chromosomes of *L. narbonense*. The site of 35S rDNA was localized in the pericentromeric region of the long arm of chromosome 1, and the site of 5S rDNA was revealed in the distal region of the long arm of chromosome 3 (Fig. 2c, d).

The cultivated species *L. usitatissimum* as well as its wild ancestor *L. angustifolium* were mesotetraploids with 2n = 30 and had significantly smaller sizes of chromosomes (1.2-3 μm) compared to the species mentioned above. The only site of 35S rDNA was observed in the proximal region of the short arm of metacentric chromosome 1. Sites of 5S rDNA were found in the proximal region of the long arm of chromosome 3 and also in the distal regions of long arms of chromosomes 8 and 13 (Fig. 2e, f).

**Fig. 3:**
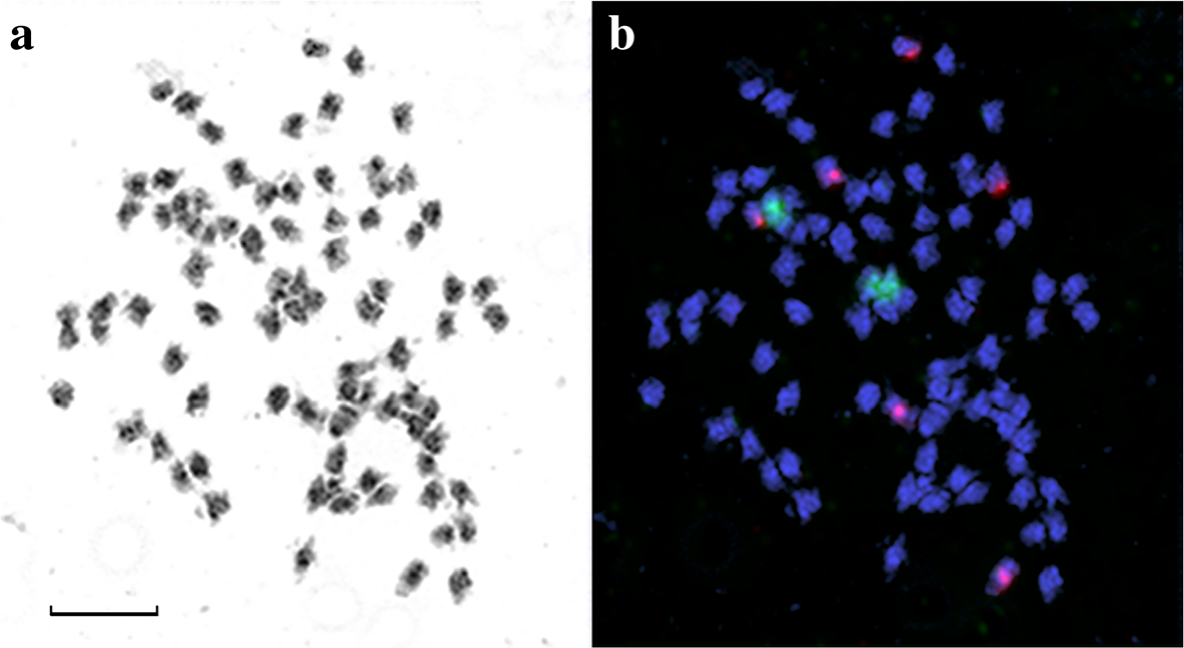
Metaphase plate of *L. marginale* 2n = 84. **a** – inverted DAPI staining of chromosomes (grey). **b** – FISH with labeled 35S (green) and 5S (red) rDNA probes. Scale bar – 5 μM

*L. marginale* (2n = 84) endemic to Australia was the last studied member of the section *Linum.* The chromosomes of this species were very small (0.7-1.5 μm). The only site of 35S rDNA was localized in one chromosome pair, and three chromosome pairs bore sites of 5S rDNA (Fig. 3).

### Structure and intragenomic variability of rDNA repeats

The PCR amplified region of 35S rRNA gene involved 33 bp of 5’end of 18S rRNA gene, ITS1 sequence, the sequence coding 5.8S rRNA gene, and ITS2 sequence (Additional file 2 **)**. It was found that intraspecific variability of the sequences of intergenic spacers ITS1 and ITS2 was significantly higher than the variability of coding regions of 5.8S rRNA gene, and it was caused mostly by single nucleotide substitutions (SNPs) and indels. Mutations of more extended regions of the sequences occurred rarely with less than 1% frequency. The analysis of the SNPs showed that the number of transitions exceeded the number of transversions. The clustering of ITS1-5.8S rDNA-ITS2 sequences revealed one major cluster including more than 5% of reads for each studied sample.

The PCR amplified sequences of 5S rRNA genes contained 98 bp of 5′ end of coding region, intergenic spacer sequence and 22 bp of 3′ end of coding region. In all studied specimens, the length of coding regions of 5S rRNA genes was 120 bp, and the length of intergenic spacers of 5S rRNA genes ranged from 136 to 239 (Additional file 3). Intraspecific variability of the spacer sequences was significantly higher than the variability of the coding regions. The variability was caused mostly by SNPs, indels and also by deletions or duplications of more extended regions of spacer sequences. The analysis of the SNPs showed that the number of transitions far exceeded the number of transversions (Additional file 4). For 5S rRNA sequences, the number of clusters per sample varied from 1 to 7. The analysis of the nature of mutations that defined clusters showed that the SNPs, in particular C➝T and G➝A transitions, were the most frequent polymorphisms in the majority of the specimens with the exception of the members of the section *Dasylinum* in which the variability of spacer sequences of 5S rRNA genes were caused mostly by deletions of different spacer regions and also by replacement of CGGAATGGGAA sequence in position 309-319 to TAAAATAATAT sequence (a subrepeat of the spacer) in all studied members of the section *Dasylinum.*

### Interspecies variability of rDNA repeats

Comparison of ITS1-5.8S rDNA-ITS2 sequences showed that 5.8S rDNA sequences were rather conservative in all studied flax specimens (Average Evolutionary Divergence 2,92 + 1,18) whereas strong sequence divergence was found in ITS1 and ITS2 sequences (Average Evolutionary Divergence for ITS1and ITS2: 35,19 + 2,49 and 34,25 + 3,14, respectively). The ITS1 and ITS2 sequences were more similar to each other inside the groups of species having similar karyotype structure.

In all studied species, the coding sequences of 5S rDNA genes were similar. They were also similar to *L. usitatissimum* sequences described earlier [26–28]. The coding sequence of flax 5S rRNA genes, in common with the sequences of other plants studied earlier, contained a highly conserved regulatory region known as the Intragenic Control Region that included A and C boxes [29, 30]. In general, the coding sequence of flax 5S rDNA genes common to blue-flowered flax species differed by only four nucleotide replacements from the consensus sequence of angiosperms [28]. In contrary, nontranscribed spacers of 5S rDNA genes were highly variable. Average Evolutionary Divergence for the spacers were 41,52± 2,13 compared to 0,78 ± 0,15 for the coding region. Similar to ITS regions, spacers of 5S rDNA genes were more similar to each other inside the groups of species having similar karyotype structure. It has been established that, in many plants, upstream and downstream spacer regions of coding sequence of 5S rRNA gene were conservative as the regulatory elements important to transcriptional mechanisms were localized there [31, 32].

In the studied flax species, these spacer regions were also conservative. So, for example, for 54 bp of the alignment upstream of the coding region, Average Evolutionary Divergence was 6.75 + 1.12; also, in four position, their nucleotide composition was similar in all group of reads of all the samples (Additional file 3). Besides, all examined samples possessed a poly-C sequence followed by a poly-T structure immediately downstream of the 5S gene coding region.

### Phylogenetic reconstruction

**Fig. 4:**
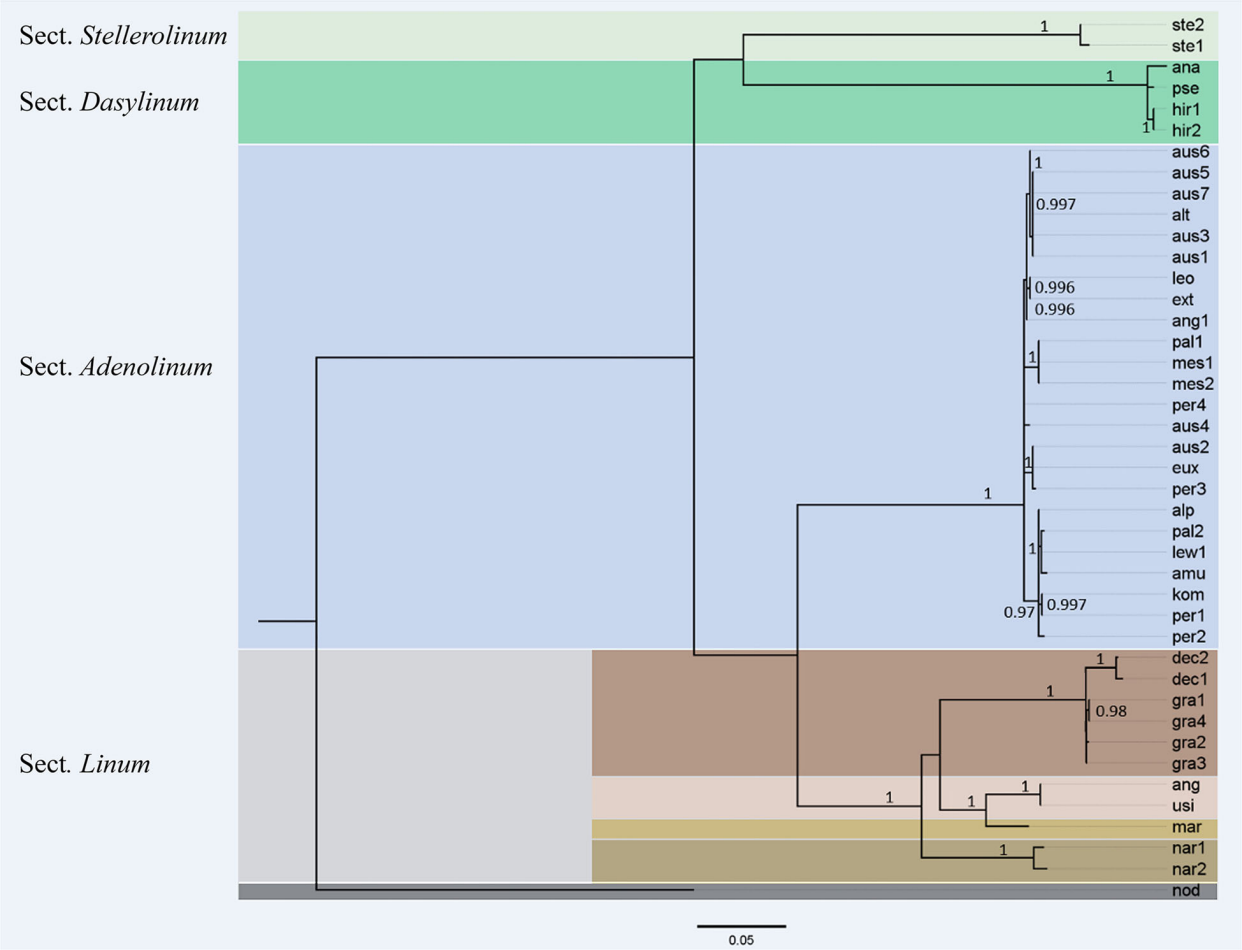
ML phylogeny of ITS1-5.8S rDNA-ITS2 consensus sequences from blue-flowered flaxes and the selected out-group – yellow-flowered flax *L. nodiflorum* (sect. *Syllinum*). The values shown below branches are node supports assessed by the Bayesian-like modification of the approximate likelihood ratio test (aBayes) [25]. The abbreviations of the names of the samples are explained in the Additional file 1

**Fig. 5:**
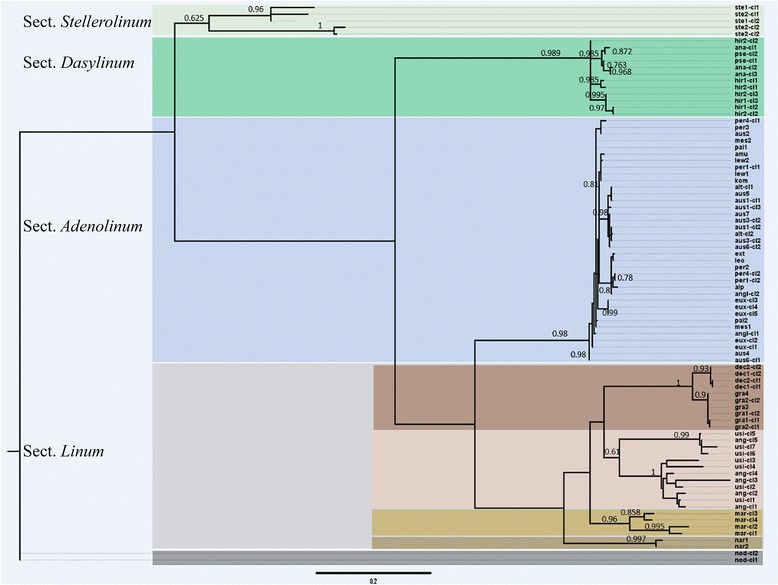
ML phylogeny of 5S rRNA genes consensus sequences from blue-flowered flaxes and the selected out-group – yellow-flowered flax *L. nodiflorum* (sect. *Syllinum*). The values shown below branches are node supports assessed by the Bayesian-like modification of the approximate likelihood ratio test (aBayes) [25]. The abbreviations of the names of the samples are explained in the Additional file 1. For flax specimens possessing different classes of 5S rRNA genes sequences, the numbers of corresponding classes (cl) are shown below the name abbreviations

**Fig. 6:**
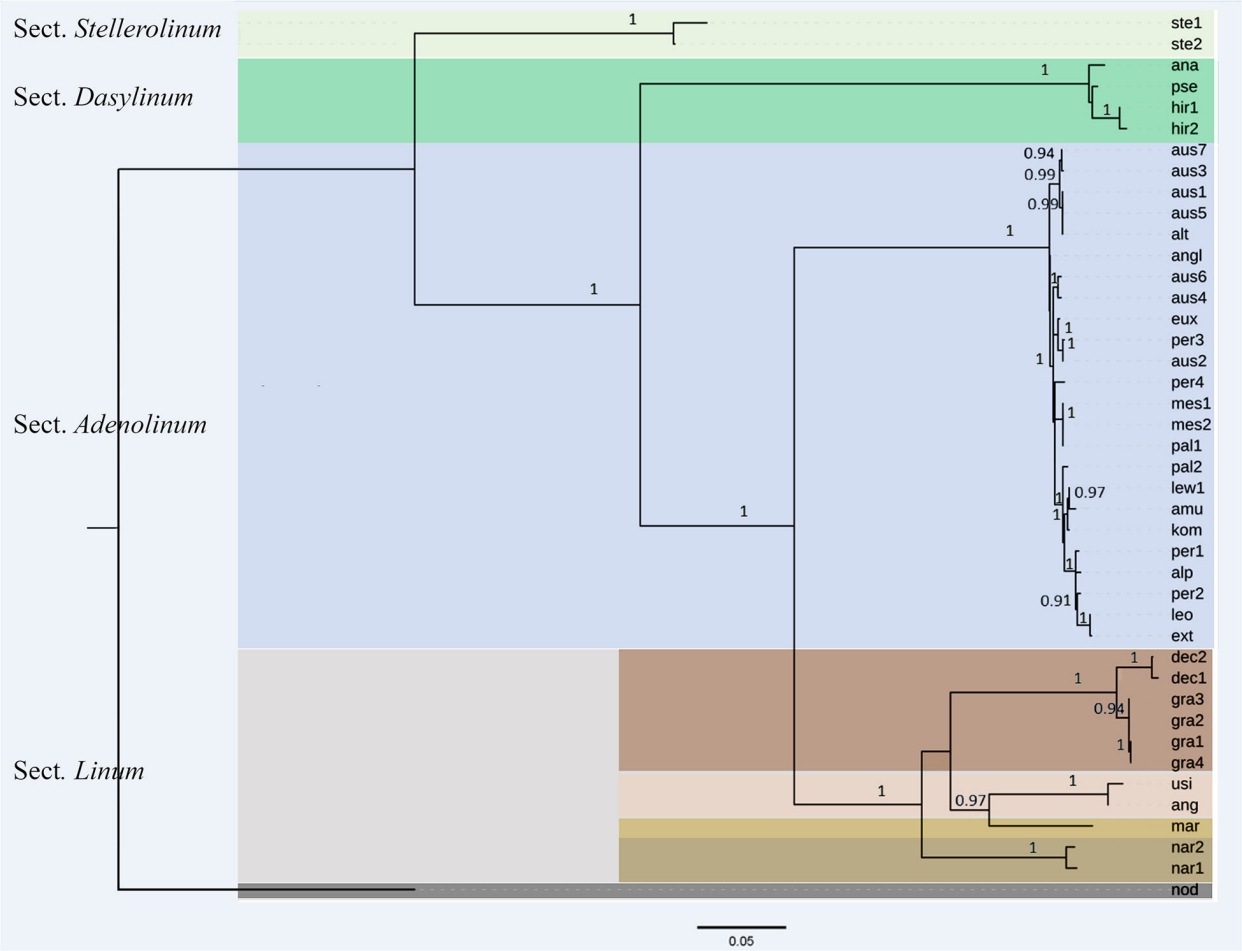
ML phylogeny of 5S rRNA genes combined with ITS1-5.8S rDNA-ITS2 consensus sequences of blue-flowered flaxes and the selected out-group – yellow-flowered flax *L. nodiflorum* (sect. *Syllinum*). The values shown below branches are node supports assessed by the Bayesian-like modification of the approximate likelihood ratio test (aBayes) [25]. The abbreviations of the names of the samples are explained in the Additional file 1

Based on the results of the sequence analysis of ITS1-5.8S rDNA-ITS2 и 5S rDNA, the ML phylogenetic trees were constructed (Figs. 4, 5). The topology of both trees was similar to each other. However, some differences were detected in the position of the branch formed by specimens of *L. stelleroides* belonging to the monotype sect. *Stellerolinum.* In the 5S rDNA tree, this species formed a basal branch of the blue-flowered clade. Upward this branch, a highly supported lineage composed of the members of sect. *Dasylinum* is set. In contrast, in the ITS1-5.8S rDNA-ITS2 tree, the members of sect. *Stellerolinum* and sect. *Dasylinum* were clustered together and formed two subclades of the basal branch of the tree, but this branching this branching was lowly supported whereas the basal position of *L. stelleroides* in the combined tree of both rDNA sequences (Fig. 6) was highly supported. Inside the sect. *Dasylinum* clade, the tetraploids *L. hirsutum* subsp. *pseudoanatolicum* and *L. hirsutum* subsp. *anatolicum* were similar but they were slightly different from the diploid *L. hirsutum* subsp. *hirsutum* according to the both classes of rDNA sequences. Finally, the branches formed by the members of the sect. *Adenolinum* and *Linum* were subdivided. Within the members of the sec. *Adenolinum*, some differences in the examined sequences were revealed; however, the specimens representing one species were not clustered together but interspersed with the specimens of the other species. In both phylogenetic trees, the members of the sect. *Linum* were subdivided into four branches. The first one is formed by the specimens of autotetraploid *L. narbonense* (2n = 4× = 28), the second branch was formed by diploid *L. decumbens* and *L. grandiflorum* (2n = 2× = 16), the third one was formed by allotetraploid cultivated *L. usitatissimum* and its wild ancestor *L. angustifolium* (2n = 4× = 30) and the fourth branch was formed by the multipolyploid Australian species *L. marginale*. Besides, in accordance with ITS phylogeny, the subcluster formed by the specimens of *L. marginale* was a sister (with high support value) to the subcluster formed by *L. usitatissimum* and *L. angustifolium*. In the phylogeny of 5S rDNA, the position of *L. marginale* was unclear as the support value was very low.

## Discussion

The study of the phylogenetic relationships within the clade of blue-flowered flaxes is especially important as it includes widely cultivated species *L. usitatissimum.* Currently, the genome of flax has been sequenced, and continued to be intensively investigated. [33–47]. However, the origin of the genome of cultivated flax is still remained uncleared.

### Evolution of karyotypes

The karyotype structure is one of the significant taxonomic characters. It was found that within the section, the flax species have the similar karyotypes (excepting the members of the sect. *Linum*) but the members of different sections vary considerably in chromosome number and morphology. The differences in chromosome number can be caused by ascending/descending aneuploidy or chromosome fusions. These phenomena are involved into the process of evolution of karyotypes of blue-flowered flaxes. It was believed that genome duplication of the ancient ancestor of the blue-flowered clade took place about 30 million years ago [9]. Perhaps, the polyploid origin of blue-flowered flaxes enabled them to loss chromosomes and to maintain the vitality during genome evolution. The intercalary bands of telomere repeats revealed in karyotypes of several species [17, 48] indicate that chromosome fusions, could also occur during the evolution of flax. Since the event of duplication of the ancestral genome, the significant diploidization of genomes has occurred, and most modern blue-flowered flaxes look like diploid. At the same time, the karyotypes of *L. hirsutum* ssp. *anatolicum*, *L. hirsutum* ssp. *pseudoanatolicum* (2n = 32), *L. perenne* ssp. *anglicum*, *L. perenne* ssp. *extraailare*, *L. perenne* k5500 (2n = 36) and *L. narbonense* (2n = 28) possess two similar diploid chromosome sets indicating that relatively recently, another duplication of their genomes has taken place. This is not surprising as polyploidization occurring by duplication of chromosomal set is widespread in angiosperms. In plant systematics, the forms having similar morphological traits but differed in ploidy levels are considered to be the cytotypes of one biological species. Changes in ploidy status lead to the whole or partial reproductive isolation, and therefore, in some cases, the appearance of cytotypes is probably the starting point of speciation [49].

The investigation of the cultivated flax (*L. usitatissimum*, 2n = 30) showed that this species is also tetraploid. The degree of divergence of the orthologous genes indicated that the origin time of this tetraploid was 5-9 million years [35, 50]. However, unlike the tetraploid species mentioned above, its chromosome set could not be subdivided in two parental genomes though similar features between several pairs of chromosomes were detected [5]. These findings indicate that either multiple chromosomal rearrangements have occurred since the event of duplication of the genome of cultivated flax or the tetraploid flax ancestor could appear as the result of distant hybridization between two diploid species having different karyotypes. Perhaps, *L. marginale* having a considerable number of chromosomes (2n = 84) is a high-ploidy-level species, but the detailed analysis of its karyotype is impossible since its chromosomes are very small.

FISH analysis showed that most studied species possessed single 5S and 35S rDNA loci in their haploid chromosome sets with the exception of members of the sect. *Dasylinum* having two 35S rDNA loci and *L. narbonense* with two 5S rDNA loci in their haploid chromosome sets. Probably, these additional loci appeared de novo during evolution but they also could result from ancient polyploidization. The tetraploid species *L. angustifolium* and *L. usitatissimum* and also polyploid *L. marginale* have one pair of satellite chromosomes in their karyotypes. One NOR is often observed in polyploid genomes of allopolyploids as a consequence of nucleolar dominance, and therefore, *L. angustifolium*, *L. usitatissimum* and *L. marginale* have probably hybrid origins. Besides, karyotypes of *L. angustifolium* and *L. usitatissimum* possess three pairs of 5S rDNA loci. It is likely that one of the parental species had two 5S rDNA loci or the additional locus appeared later as a result of dispersion of 5S rDNA throughout the genome. Interestingly, we detected only three pairs of 5S rDNA loci in multipolyploid *L. marginale* (2n = 84). The other 5S rDNA repeats were probably lost during formation of the polyploid genome, and they could not be revealed by FISH. We previously observed the same phenomenon in some yellow-flowered flaxes of sect. *Sillinum* [17].

### Variability and evolution of rRNA genes

The reported here ITS1-5.8S rDNA-ITS2 sequences are in agreement with the sequences obtained early by the cloning method [8]. In the studied flaxes similar to many other plants and animals [51], low levels of intraspecific variability of ITS1-5.8S rDNA-ITS2 sequences revealed, and the character of their variability agreed with the model of concerted evolution. It is believed that high levels of homogeneity of these repeats provides the presence of a quality control system that prevents the accumulation of rDNA mutations [52–54]. The results of high-throughput sequencing for the species of the genus *Nicotiana* revealed correlation between the number of 35S rDNA loci and variability of ITS1-5.8S rDNA-ITS2 sequences [55]. However, we did not find a significant increase in variability of these sequences within diploid and tetraploid members of the sect. *Dasylinum* having two NOR loci in a haploid set as well as in tetraploids from the sect. *Adenolinum* and *L. narbonense* from the sect. *Linum* bearing active NOR in each of their four subgenomes compared to the other species having one NOR pair. It is quite possible that this discrepancy between the members of *Nicotiana* and *Linum* was due to the fact that multiple NOR loci in *Linum* could formed by the duplication of a single loci of the ancestor species and therefore, they were initially similar. In the present study, the high-throughput sequencing was used for the first time for analysis of 5S rRNA genes. In flax, the intraspecific variability of the spacers of 5S rRNA genes was considerably higher than the variability of ITS1 and ITS2 sequences of 35S rRNA genes. The highest variability was found in *L. angustifolium*, *L. usitatissimum* and *L. stelleroides*. The heterogeneity of 5S rRNA genes was observed earlier in a lot of other species [56]. Unlike 35S rRNA genes (their variability is usually in good agreement with the model of concerted evolution), the type of variation of 5S rRNA genes often agrees with the model of birth-and-death evolution or with the mixed process of concerted evolution and birth-and-death evolution [51, 57, 58].

It was supposed that the newly generated duplicate genes or gene families may evolve to interact with other existing gene families and promote the adaptation of organisms to new environments [51].

Despite the question if the heterogeneity of 5S rRNAs is somehow associated with their functions remains unresolved in most cases, there is a great deal of evidence that different variants of 5S rRNA genes could be differentially expressed during development [59–61]. In *Arabidopsis thaliana*, different variants of 5S rRNA were found in the cells of seeds and roots [62].

### Phylogenetic reconstruction of blue-flowered flaxes

It should be highlighted that the results of phylogenetic analyses based on the 5S rDNA and ITS sequences were analogous with each other. They were in good agreement with the phylogenetic studies reported earlier [8–10, 63] and also with the results of the comparative katyotypic analyses performed in the present work. Particularly, according to our results, the topology of the tree constructed with the use of ITS sequences almost coincided with the topology described earlier [8]. The results of phylogenetic analyses reported here are agreed with the type of variation of some morphological traits revealed among the members of different sections. Particularly, in *L. stelleroides*, margins of sepals are membranous with large black stipitate glands. The members of the sect. *Dasylinum* have glandular-ciliate sepals and stipitate glands are small and white. Most species of the sect. *Adenolinum* and *Linum* have eglandular sepals with the exception of *L. nervosum* Waldst. et Kit (not examined in this study) which margins of sepals are slightly serrulate-ciliate with small short stalked glands. Interestingly, for a long time, *L. nervosum* was considered to be a primitive member of the sect. *Linum* [8] but molecular phylogenetic studies [8] showed that this species represented a basal branch of the clade formed by the members of the sect. *Adenolinum*. Sepals of the other members of the sect. *Adenolinum* have entire, scarious margins. The species of the sect. *Linum* possess sepals with ciliate margins. Molecular phylogenetic studies also indicated that the *L. narbonense* was the most ancient species among the members of the sect. *Linum.* This species has sepals with minutely serrulate-ciliate, scarious margins. Сonsidering the fact that secretome of sepal glands attracts pollinating insects, there is probably any relationship between the evolution of sepals in flax and a species composition of the pollinating insects though this problem is not adequately investigated.

Besides, there is a tendency for increase of sizes of seeds and seed capsuls among the sect. *Stellerolinum - Dasylinum - Adenolinum* - *Linum* with the exception of *L. marginale* which has small seeds. It is important to note that the structure of pollen grains in the most ancient species *L. stelleroides* differs greatly compared to the other species. *L. stelleroides* has pantoporate pollen grains with 12 pores whereas the members of the other three sections have tricolpate pollen with the exception of few popyploid species having polycolpate pollen [64]. The relationship between the revealed phylogeny and geographical distribution of the species is not quite clear. The centre of origin of genus *Linum* was believed to locate in the Mediterranean region and western Asia where the most species diversity of wild flaxes of the Old World is observed [8]. Inside this region, the areas of distribution of the separate sections are mostly overlapped. However, the areas of distribution of the evolutionary advanced species, such as members of the sect. *Adenolinum* and also *L. angustifolium* and *L. usitatissimum* from the sect. *Linum*, are wider and spread farther to the North. The progenitor species of Australian *L. marginale* and its close relative from New Zealand *L. monogynum* Rchb. (sect. *Linum*) were probably arrived from the Northern Hemisphere about 5-1 mya similar to many other members of other families of angiosperms [65]. Interestingly, among the species originated in Northern Hemisphere and migrated to Australia and New Zealand regions, a great number of polyploids including the studied *L. marginale* and also *L. monogynum* (2n = 84) were revealed. Unlike the most species of the Old World, the oldest member of blue-flowered flaxes, *L. stelleroides*, grows in East Asia. Probably, the progenitor of *L. stelleroides* was distributed across the entire Asia region, but it could survive to nowadays only in the east where where it ‘had waited out’ glaciations in China refugia. Alternatively, the Mediterranean region and western Asia were not the primary but secondary centre of origin of blue-flowered flaxes, and the primary centre was located much farther east than it was thought.

Unlike the previous studies [8–10, 63], we used more species and specimens of the sect. *Adenolinum* and this allowed us to examine the relationships within this section in greater detail. We detected that the specimens belonging to one and the same species were not always clustered with each other but interspersed with the specimens of other species. Besides, in the present study, the full ITS sequence for *L. narbonense* (sect. *Linum*) was obtained, and this allowed us to clarify phylogenetic relationships of this species. It was found that this species was clustered with the other members of the sect. *Linum* despite the considerable karyotypic differences.

The sequences of 5SrRNA genes were successfully used in phylogenetic analyses of various groups of plants and animals. Along with this, a number of variants of 5S rRNA genes differed in DNA sequences were revealed in genomes of many organisms [56]. In particular, we revealed this phenomenon in *L. usitatissimum.* High variability of the sequences of 5S rRNA genes in *L. usitatissimum* was also described early [28].

Besides, we revealed that in most studied species intraspecific variability of the sequences of 5S rRNA genes was usually less than interspecific variability with the exception of cultivated species *L. usitatissimum* and its wild ancestor *L. angustifolium* from the sect. *Linum* as well as the members of sect. *Adenolinum*.

According to the obtained ITS phylogeny and the phylogeny based on the mitochondrial rcbL [8], *L. stelleroides* was clustered with the members of the sect. *Dasyllinum*. At the same time, in accordance with the obtained phylogeny of 5S rDNA as well as the phylogeny based on chloroplast (*ndhF* + *trnK* intron + *trnL-F*) and also ITS phylogenies [8], *L. stelleroides* formed a basal clade which was independent from the clade of sect. *Dasyllinum*. These data and also the detected karyological and morphological differences [2] indicate that *L. stelleroides* is not a close relative to the species of the sect. *Dasyllinum.*

As already mentioned, using 5S rDNA or ITS sequences as well as different DNA fingerprinting methods [4, 7, 16] did not allow us to reveal the specific differences within the sect. *Adenolinum.* Besides, these species were also difficult to distinguish morphologically. The classical taxonomical treatment of this group is based on the quantitative characters which are unstable and depend on the environmental conditions [11, 66]. As up to the present moment the stable and also taxonomically significant characters, which are adequate for speciation within this group, have not been revealed. Therefore, we consider the members of the sect. *Adenolinum* as a single highly polymorphic species widespread in the North Hemisphere. Interestingly, the same treatment of *L. perenne* group (syn. sect. *Adenolinum*) was proposed by Planchon as early as in 1848 (cited according to [11]). The taxonomic classification of the species *L. narbonense* from the sect. *Linum* was controversial as this species differs considerably in chromosome number and sizes, chromosomal distribution of rDNA sites and also due to a low level of similarity with EST-SSRs sequences from the other members of the section, particularly, from *L. usitatissimum* [67]. However, according to our results, phylogenies of 5S rDNA and ITS as well as the phylogeny based on the mitochondrial rbcL genes [8] this species is clustered significantly with the other members of the sect. *Linum*.

### Phylogeny of *L. usitatissimum* and its possible origin

The cultivated flax (*L. usitatissimum*, 2n = 30) was shown to be an allotetraploid species [35, 50]. Based on both 5S rDNA and ITS phylogenies, we revealed close relationships between *L. usitatissimum* and *L. angustifolium* which is considered to be a wild ancestor of the cultivated flax. Both species have identical karyotypes (2n = 30) and similar distribution of 5S and 35S rDNA sites. High degree of relationship between these species is also confirmed by the results of whole genomes and transcriptomes sequencing [9, 10, 37]. Moreover, some taxonomists do not consider them as separate species but subspecies: *L. usitatissimum* subsp*. usitatissimum* L. and *L. usitatissimum* L. subsp. *angustifolium* (Huds.) Thell. According to morphological characters and molecular phylogenetic investigations, the multipolyploid *L. marginale* (2n = 84) is relative to tetraploid *L. usitatissimum* and *L. angustifolium.* Despite the differences in ploidy levels, these species have similar chromosomal localization of 5S and 45S rRNA genes, and this allowed us to assume that their polyploid genomes could comprise common subgenomes. Unfortunately, it is impossible to verify this assumption as the genome of *L. marginale* has not been sequenced yet. Polyploid species *L. usitatissimum*, *L.angustifolium* and *L. marginale* are related to the karyologically similar diploids *L. grandiflorum* (2n = 16) and *L. decumbens* (2n = 16) and also autotetraploid *L. narbonense* (2n = 28). All these species are similar in a number of morphological traits (alternate, glabrous leaves, without stipular glands; eglandular, ciliate sepals; blue, purple, pink or white, free petals; linear stigmas) and they are included in the sect. *Linum.* Close relationship between genomes of *L. usitatissimum* and *L.angustifolium* and genomes of *L. grandiflorum* and *L. decumbens* is also confirmed by the results of whole genomes and transcriptomes sequencing [35, 50]. These data together with the revealed common structure of satellite chromosomes allowed us to suggest that the ancestor of the modern allotetraploids *L. angustifolium* and *L. usitatissimum* could appear as a result of hybridization between two ancient members of the lineage with the basic chromosome number x = 8 (a relative of modern *L. grandiflorum* and *L. decumbens*) followed by diploidization of the hybrid genome. In the genome of this newly formed allotetraploid, the reduction of chromosomes from 2n = 32 to 2n = 30 could occur during evolution. Allotetraploid with 2n = 30 could also appear after hybridization between the ancestor of modern *L. narbonense* with the basic chromosome number x = 7 and the ancestor of modern *L. grandiflorum* and *L. decumbens* with the basic chromosome number x = 8; and this hybridization led to the formation of a new chromosome number *n* = 7 + 8 = 15. An argument in favor of the second hypothesis could be the fact that the number of 5S rDNA sites in karyotypes of *L. angustifolium* and *L. usitatissimum* (three pairs) was equal to the total number observed in the karyotypes of *L. grandiflorum* or *L. decumbens* (one pair) and in the diploid karyotype of *L. narbonense* (two pairs). However, the both hypotheses obviously need further confirmations.

## Conclusions

Combined molecular and cytogenetic studies and also high-throughput sequencing of multicopy rRNA gene families allowed us to make several adjustments to the phylogeny of blue-flowered flax species and also reveal the intra- and interspecific diversity of the rRNA gene sequences. In particular, the basal position of *L. stelleroides* within the clade of blue-flowered species and also weak divergence among members of the sect. *Adenolinum* were confirmed. The specification of the phylogenetic relations among the members of the sect. *Linum* can contribute to a better understanding of the processes underlying the formation and evolution of the cultivated flax – *L. usitatissimum*.

## Additional files

Additional file 1: The studied accessions of the genus *Linum*. (DOC 99 kb)

Additional file 2: Consensus ITS1-5.8S rDNA-ITS2 sequences of the studied flax samples. (TXT 27 kb)

Additional file 3: Consensus sequences of 5S rRNA genes of the studied flax samples. For flax specimens possessing different classes of 5S rRNA genes sequences, the numbers of corresponding classes (cl) are shown below the name abbreviations. (TXT 31 kb)

Additional file 4: Intragenomic variability of 5S rRNA genes. (DOC 108 kb)
