# Supplementary material for: Evolution of blue-flowered species of genus Linum based on high-throughput sequencing of ribosomal RNA genes
Source: BMC Evol Biol. 2017 Dec 28;17(Suppl 2):253. doi: 10.1186/s12862-017-1105-x (PMC5751768; doi:10.1186/s12862-017-1105-x)
Supplement: Supplementary file 1 — The studied accessions of the genus Linum. (DOC 99 kb) [file 12862_2017_1105_MOESM1_ESM.doc]

**Table S1. The studied accessions of genus *Linum*.**

| **N** | **Accession name** | **Source** | **Genebank**  **number** | **Origin** | **Abbr.** |
| --- | --- | --- | --- | --- | --- |
| Sect. *Adenolinum* | | | | | |
| 1 | *L. perenne* L. | IPK | LIN 1807 | Russian Federation | per1 |
| 2 | *L. perenne* L. subsp.  *extraaxillare* (Kit.)  Nyman | IPK | LIN 1651 | Poland | ext |
| 3 | *L. altaicum* Ledeb.  ex Juz. | IPK | LIN 1632 | unknown | alt |
| 4 | *L. komarovii* Juz. | IPK | LIN 1716 | unknown | kom |
| 5 | *L. perenne* L. | IPK | LIN 1521 | Slovakia | per2 |
| 6 | *L. perenne* L. | Wild population,  collected by  A.A. Svetlova | - | Russian Federation,  Rostov region,  village Nesvetai  neighborhood | per3 |
| 7 | *L. perenne* L. subsp.  *alpinum* (Jacq.)  Stoj. & Stef. | IPK | LIN 1905 | Austria | alp |
| 8 | *L. perenne* L. subsp.  *anglicum* (Mill.)  Ockendon | IPK | LIN 1524 | GB | angl |
| 9 | *L. perenne* L. | VNIIL | K 5500 | unknown | per4 |
| 10 | *L. leonii* F.W.Schultz | IPK | LIN 1672 | Germany | leo |
| 11 | *L. pallescens* Bunge | IPK | LIN 1645 | Tajikistan | pal1 |
| 12 | *L. pallescens* Bunge | Wild population,  collected by  N. L. Bolsheva | - | Russian Federation,  Altai | pal2 |
| 13 | *L. mesostylum* Juz. | IPK | LIN 1774 | Tajikistan | mes1 |
| 14 | *L. mesostylum* Juz. | IPK | LIN 1662 | Tajikistan | mes2 |
| 15 | *L. lewisii* Pursh | IPK | LIN 1648 | USA | lew1 |
| 16 | *L. lewisii* Pursh | IPK | LIN 1550 | USA | lew2 |
| 17 | *L. austriacum* L. | IPK | LIN 1608 | Germany | aus1 |
| 18 | *L. austriacum* L. | Wild population,  collected by  A.A. Sveatlova | - | Russian Federation,  Rostov | aus2 |
| 19 | *L. austriacum* L.  subsp. *euxinum* (Juz.)  Ockendon | IPK | LIN 1546 | Ukraine | eux |
| 20 | *L. austriacum* L. | Wild population,  collected by  A.A. Svetlova | - | Crimea | aus3 |
| 21 | *L. austriacum* L. | Wild population,  collected by  A.A. Svetlova | - | Ukraine,  Donezk | aus4 |
| 22 | *L. austriacum* L. | VNIIL | K 3432 | Russian Federation,  Stavropol region | aus5 |
| 23 | *L. austriacum* L. | IPK | LIN 6 | unknown | aus6 |
| 24 | *L. austriacum* L. | IPK | LIN 1831 | unknown | aus7 |
| 25 | *L. amurense* Alef. | BGI | Outdoors  cultivated  collection | Russian Federation,  Far East, near  Pokrovka settlement | amu |
| Sect. *Dasylinum* | | | | | |
| 26 | *L. hirsutum* L.  subsp. *hirsutum* L. | IPK | LIN 1676 | Hungary | hir1 |
| 27 | *L*. *hirsutum* subsp.  *hirsutum* L. | IPK | LIN 1649 | Romania | hir2 |
| 28 | *L. hirsutum* L. subsp.  *pseudoanatolicum*  P.H.Davis | Wild population,  collected by  M. Pavelka | - | Turkey, Karaman | pse |
| 29 | *L. hirsutum* L. subsp  *anatolicum* (Boiss.)  Hayek | Wild population,  collected by  M. Pavelka | - | Turkey, Aksaray | ana |
| Sect. *Linum* | | | | | |
| 30 | *L. marginale* A.Cunn.  ex Planch | IPK | LIN 1920 | Australia | mar |
| 31 | *L. narbonense* L. | IPK | LIN 2002 | unknown | nar1 |
| 32 | *L. narbonense* L. | IPK | LIN 1653 | France | nar2 |
| 33 | *L. decumbens* Desf. | IPK | LIN 1754 | Italy | dec1 |
| 34 | *L. decumbens* Desf. | IPK | LIN 1913 | Italy | dec2 |
| 35 | *L. grandiflorum* Desf. | IPK | LIN 2000 | unknown | gra1 |
| 36 | *L. grandiflorum* Desf. | IPK | LIN 974 | unknown | gra2 |
| 37 | *L. grandiflorum* Desf. | IPK | LIN 4 | unknown | gra3 |
| 38 | *L. grandiflorum* Desf. | VNIIL | var. Scarlet |  | gra4 |
| 39 | *L. angustifolium* Huds. | IPK | LIN 1642 | East Germany | angu |
| 40 | *L. usitatissimum* L. | IPK | LIN 633 | Czechoslovakia | usi |
| Sect. *Stellerolinum* | | | | | |
| 41 | *L. stelleroides*  Planchon | BGI | Outdoors  cultivated  collection | Russian Federation,  Far East,  Telyakovsky Inlet | ste1 |
| 42 | *L. stelleroides*  Planchon | BGI | Outdoors  cultivated  collection | Russian Federation,  Far East, near  Kraskino settlement | ste2 |
| Sect. *Syllinum* | | | | | |
| 43 | *L. nodiflorum* L. | IPK | LIN 1634 | unknown | nod |
