# Supplementary material for: Evolution of blue-flowered species of genus Linum based on high-throughput sequencing of ribosomal RNA genes
Source: BMC Evol Biol. 2017 Dec 28;17(Suppl 2):253. doi: 10.1186/s12862-017-1105-x (PMC5751768; doi:10.1186/s12862-017-1105-x)
Supplement: Supplementary file 4 — Intragenomic variability of 5S rRNA genes. (DOC 108 kb) [file 12862_2017_1105_MOESM4_ESM.doc]

**Table S4. Intragenomic variability of 5S rRNA genes.**

| **N** | **Accession name** | **Number of reads** | **Length (bp)** | **GC content (%)** | **transition/transversion**  **bias (R)** | **Diversity** | | **Number of groups** |
| --- | --- | --- | --- | --- | --- | --- | --- | --- |
|  |  |  |  |  |  | **Coding gene region** | **Spacer** |  |
| Sect. *Adenolinum* | | | | | | | | |
| 1 | *L. perenne* L.  (per1) | 105 | 350 | 41.8 | 1.63 | 0.01  ±0.003 | 0.03  ±0.004 | 1 |
| 2 | *L. perenne* L. subsp.  *extraaxillare* (Kit.) Nyman  (ext) | 36 | 349 | 41.4 | 1.24 | 0.02  ±0.01 | 0.03  ±0.004 | 1 |
| 3 | *L. altaicum* Ledeb. ex Juz.  (alt) | 70 | 350 | 41.3 | 2.77 | 0.01  ±0.002 | 0.03  ±0.004 | 2 |
| 4 | *L. komarovii* Juz.  (kom) | 106 | 350 | 42 | 1.54 | 0.02  ±0.003 | 0.03  ±0.005 | 1 |
| 5 | *L. perenne* L.  (per2) | 256 | 350 | 42.2 | 1.14 | 0.01  ±0.002 | 0.01  ±0.002 | 1 |
| 6 | *L. perenne* L.  (per3) | 218 | 350 | 42.0 | 1.51 | 0.01  ±0.002 | 0.02  ±0.003 | 1 |
| 7 | *L. perenne* L. subsp. *alpinum*  (Jacq.) Stoj. & Stef.  (alp) | 229 | 350 | 42.1 | 1.54 | 0.008  ±0001 | 0.02  ±0.002 | 1 |
| 8 | *L. perenne* L. subsp. *anglicum*  (Mill.) Ockendon  (angl) | 241 | 350 | 41.6 | 1.51 | 0.01  ±0.002 | 0.03  ±0.003 | 2 |
| 9 | *L. perenne* L.  (per4) | 61 | 350 | 41.9 | 1.65 | 0.01  ±0.002 | 0.04  ±0.008 | 2 |
| 10 | *L. leonii* F.W.Schultz  (leo) | 191 | 349 | 41.5 | 1.13 | 0.01  ±0.003 | 0.03  ±0.004 | 1 |
| 11 | *L. pallescens* Bunge  (pal1) | 171 | 350 | 42.3 | 1.59 | 0.017  ±0.002 | 0.027  ±0.004 | 1 |
| 12 | *L. pallescens* Bunge  (pal2) | 294 | 350 | 42.4 | 1.13 | 0.017  ±0.003 | 0.030  ±0.004 | 1 |
| 13 | *L. mesostylum* Juz.  (mes1) | 138 | 350 | 42.5 | 1.55 | 0.016  ±0.003 | 0.024  ±0.004 | 1 |
| 14 | *L. mesostylum* Juz.  (mes2) | 196 | 350 | 42.5 | 1.50 | 0.015  ±0.002 | 0.023  ±0.003 | 1 |
| 15 | *L. lewisii* Pursh  (lew1) | 149 | 351 | 42.5 | 1.22 | 0.013  ±0.002 | 0.015  ±0.002 | 1 |
| 16 | *L. lewisii* Pursh  (lew2) | 44 | 350 | 42.1 | 1.10 | 0.012  ±0.002 | 0.013  ±0.005 | 1 |
| 17 | *L. austriacum* L.  (aus1) | 265 | 350 | 41.4 | 1.68 | 0.007  ±0.001 | 0.018  ±0.004 | 3 |
| 18 | *L. austriacum* L.  (aus2) | 347 | 350 | 42.2 | 1.64 | 0.009  ±0.001 | 0.015  ±0.002 | 1 |
| 19 | *L. austriacum* L. subsp.  *euxinum* (Juz.) Ockendon  (eux) | 184 | 350,  341,  339,  339,  350 | 43.0 | 1.27 | 0.005  ±0.001 | 0.031  ±0.006 | 5 |
| 20 | *L. austriacum* L.  (aus3) | 113 | 350 | 41.9 | 1.43 | 0.013  ±0.002 | 0.019  ±0.003 | 2 |
| 21 | *L. austriacum* L.  (aus4) | 249 | 349 | 42.2 | 1.25 | 0.009  ±0.001 | 0.020  ±0.004 | 1 |
| 22 | *L. austriacum* L.  (aus5) | 166 | 350 | 41.4 | 1.68 | 0.007  ±0.001 | 0.018  ±0.004 | 1 |
| 23 | *L. austriacum* L.  (aus6) | 105 | 349,  350 | 41.8 | 1.24 | 0.007  ±0.001 | 0.034  ±0.006 | 2 |
| 24 | *L. austriacum* L.  (aus7) | 137 | 350 | 41.4 | 1.70 | 0.010  ±0.002 | 0.012  ±0.001 | 1 |
| 25 | *L. amurense* Alef.  (amu) | 55 | 349 | 41.7 | 1.72 | 0.007  ±0.004 | 0.046  ±0.008 | 1 |
| Sect. *Dasylinum* | | | | | | | | |
| 26 | *L. hirsutum* L. subsp.  *hirsutum* L.  (hir 1) | 111 | 351,  342,  333 | 51.1 | 1.40 | 0.012  ±0.005 | 0.034  ±0.007 | 3 |
| 27 | *L*. *hirsutum* subsp.  *hirsutum* L.  (hir2) | 227 | 353,  359,  351,  330 | 46.9 | 1.33 | 0.009  ±0.003 | 0.056  ±0.010 | 4 |
| 28 | *L. hirsutum* L. subsp.  *pseudoanatolicum* P.H.Davis  (pse) | 62 | 349 | 48.7 | 1.48 | 0.008  ±0.006 | 0.085  ±0.053 | 2 |
| 29 | *L. hirsutum* L. subsp.  *anatolicum* (Boiss.)  (ana) | 57 | 347,  351,  344 | 46.4 | 1.33 | 0.010  ±0.005 | 0.045  ±0.029 | 3 |
| Sect. *Linum* | | | | | | | | |
| 30 | *L. marginale*  A.Cunn. ex Planch  (mar) | 53 | 397,  311,  335,  334 | 45.6 | 1.19 | 0.002  ±0.002 | 0.119  ±0.015 | 4 |
| 31 | *L. narbonense* L.  (nar1) | 88 | 357 | 44.7 | 0.96 | 0.042  ±0.008 | 0.084  ±0.016 | 1 |
| 32 | *L. narbonense* L.  (nar2) | 120 | 358 | 39.3 | 1.04 | 0.049  ±0.006 | 0.104  ±0.009 | 1 |
| 33 | *L. decumbens* Desf.  (dec1) | 142 | 320,  325 | 47.6 | 1.04 | 0.021  ±0.003 | 0.033  ±0.005 | 2 |
| 34 | *L. decumbens* Desf.  (dec2) | 106 | 320,  326 | 47.5 | 1.17 | 0.020  ±0.004 | 0.045  ±0.007 | 2 |
| 35 | *L. grandiflorum* Desf.  (gra1) | 277 | 317,  296 | 46.6 | 1.57 | 0.008  ±0.001 | 0.010  ±0.001 | 2 |
| 36 | *L. grandiflorum* Desf.  (*gra2)* | 358 | 296,  316 | 46.5 | 1.28 | 0.008  ±0.001 | 0.012  ±0.002 | 2 |
| 37 | *L. grandiflorum* Desf.  (gra3) | 236 | 317 | 46.7 | 1.61 | 0.010  ±0.002 | 0.010  ±0.001 | 1 |
| 38 | *L. grandiflorum* Desf.  (gra4) | 328 | 317 | 46.7 | 1.61 | 0.010  ±0.001 | 0.016  ±0.002 | 1 |
| 39 | *L. angustifolium* Huds.  (ang) | 408 | 348,  347,  330,  341,  344 | 47.5 | 0.83 | 0.010  ±0.001 | 0.016  ±0.002 | 5 |
| 40 | *L. usitatissimum* L.  (usi) | 223 | 46,  341,  328,  325,  344,  345,  344 | 47.2 | 0.79 | 0.043  ±0.011 | 0.195  ±0.025 | 7 |
| Sect. *Stellerolinum* | | | | | | | | |
| 41 | *L. stelleroides* Planchon  (ste1) | 243 | 259,  256,  257 | 48.6 | 1.30 | 0.044  ±0.006 | 0.248  ±0.037 | 3 |
| 42 | *L. stelleroides* Planchon  *(*ste2) | 109 | 260,  258 | 48.2 | 1.34 | 0.052  ±0.010 | 0.248  ±0.048 | 2 |

*Note:* Transition/Transversion bias (*R*) was estimated under the Tamura model . For sequences diversity estimation the number of base substitutions per site from averaging over all sequence pairs were determine. Standard error estimates were obtained by a bootstrap procedure (100 replicates). Analyses were conducted Kimura 2-parameter model . All positions with less than 95% site coverage were eliminated. That is, fewer than 5% alignment gaps, missing data, and ambiguous bases were allowed at any position.

**References**

1. Tamura K. Estimation of the number of nucleotide substitutions when there are strong transition-transversion and G+C-content biases. Molecular biology and evolution. 1992;9(4):678-687.

2. Kimura M. A simple method for estimating evolutionary rates of base substitutions through comparative studies of nucleotide sequences. Journal of molecular evolution. 1980;16(2):111-120.
